# Primary Leiomyosarcoma of the Mediastinum: A Rare and Challenging Diagnosis?

**DOI:** 10.3390/diagnostics12112581

**Published:** 2022-10-25

**Authors:** Massimiliano Mancini, Gabriele Masselli, Roberto Cirombella, Renato Covello, Gianfranco Gualdi, Antonio D’Andrilli, Erino Angelo Rendina, Andrea Vecchione

**Affiliations:** 1 Morphological and Molecular Pathology Unit, Sant’Andrea Hospital, 00189 Rome, Italy; 2 Department of Radiological Sciences, Oncology, and Anatomic-Pathology, “Sapienza” University of Rome, 00185 Rome, Italy; 3 Department of Clinical and Molecular Medicine, Faculty of Medicine and Psychology, Sant’Andrea Hospital, “Sapienza” University of Rome, 00185 Rome, Italy; 4 Department of Pathology, IRCCS, Regina Elena National Cancer Institute, 00144 Rome, Italy; 5 PET/CT Section, Pio XI Private Hospital Rome, 00165 Rome, Italy; 6 Thoracic Surgery Unit, Sant’Andrea Hospital, “Sapienza” University of Rome, 00185 Rome, Italy

**Keywords:** leiomyosarcoma, primary mediastinum sarcoma, core needle biopsy

## Abstract

(1) Introduction: Leiomyosarcomas are highly aggressive mesenchymal neoplasm derived from smooth muscle cells which, in the mediastinum, are present in various primary organs; To our knowledge, less than 10 cases of primary mediastinal leiomyosarcoma have been described. Here, we report a compelling case of primary mediastinal leiomyosarcoma. (2) Case presentation: A 79-year-old woman was admitted to the Thoracic Surgery Unit of S. Andrea University Hospital for persisting cough, exertional dyspnea, and sternal pain. After multidisciplinary consultation, a CT-guided core needle biopsy of the mass was performed, resulting in a provisional diagnosis of mesenchymal neoplasm with smooth muscle differentiation without apparent signs of atypia. The patient underwent surgery that revealed a large irregularly shaped mass with a whorled pattern cut surface, showing admixed yellowish areas of necrosis and areas of hemorrhage. Histologic examination showed a smooth muscle neoplasm with atypia and necrosis, and a grade 2 primary mediastinal leiomyosarcoma diagnosis was given. (3) Conclusions: Soft tissue sarcomas represent a challenging diagnostic group of tumors due to their location, morphologic spectrum, and unique molecular background. Our case of primary mediastinal leiomyosarcoma shows how tumor heterogeneity and limited tissue sampling impact diagnosis. Further studies are needed to shed light on the disease by finding an appropriate molecular signature for each leiomyosarcoma subgroup, providing a more precise diagnosis and the correct background for tailored therapy.

## 1. Introduction

Due to histologic heterogeneity, primary mediastinal sarcomas represent a challenging diagnostic entity, especially on needle biopsy samples. They comprise an infrequent group of mesenchymal neoplasms representing less than 10% of primary mediastinal tumors (Siegel 2021); leiomyosarcomas represent 5–10% of all mediastinal sarcomas. Leiomyosarcomas are highly aggressive mesenchymal neoplasm derived from smooth muscle cells, which, in the mediastinum, are present in various primary organs such as great blood vessels, the heart, and the esophagus. Diagnostic work-up of mediastinal leiomyosarcoma involvement may be challenging, as there is a broad spectrum of differential mediastinal neoplasm to consider, including benign and metastatic diseases and conditions with similar clinical-radiological appearance. A multidisciplinary interaction between clinicians from different specialties appears crucial for an accurate diagnosis. In this context, the role of interventional radiologists and thoracic surgeons is essential to obtain adequate tissue samples, and to allow pathologists’ detection of the peculiar histological features, often unevenly distributed.

To our knowledge, less than 10 cases of primary mediastinal leiomyosarcoma have been reported (2–9); here, we report an interesting case of primary mediastinal leiomyosarcoma in a 79-year-old female.

## 2. Case Presentation

A 79-year-old woman was admitted to the Thoracic Surgery Unit of S. Andrea University Hospital for persisting cough, exertional dyspnea, and sternal pain.

**Figure 1:**
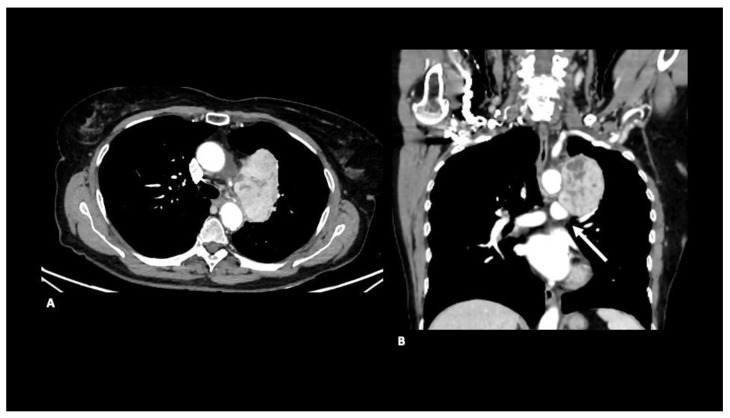
Axial (**A**) and coronal (**B**) contrast-enhanced CT images show a large, heterogeneous mediastinal mass that extends into the aortopulmonary window and compresses the left pulmonary artery. Note the large size and the heterogeneous attenuation, standard features of leiomyosarcoma.

A contrast-enhanced CT scan showed a large, heterogeneous mass extending into the aortopulmonary window and compressing the left pulmonary artery (Figure 1). 

**Figure 2:**
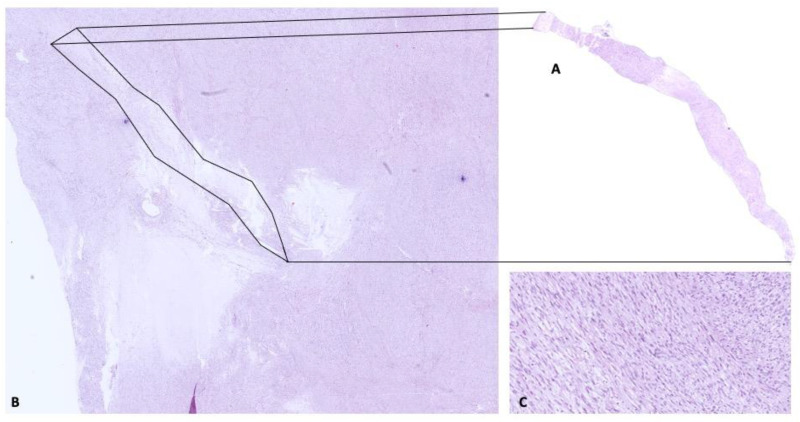
A CT-guided diagnostic core biopsy (**A**) was performed, retrieving a representative amount of tissue from the mediastinal mass. (**B**) Unfortunately, the needles punctured a low-grade area devoid of cytologic atypia, and mitotic figures CEE stain A and B (1.2 original magnification) (**C**) (20 × original magnification).

After multidisciplinary consultation, a CT-guided core needle biopsy of the mass was performed. Histological examination (Figure 2) showed a diffuse to a storiform proliferation of bland spindle cells characterized by mild nuclear atypia and dense eosinophilic cytoplasm loosely arranged in a fibrous stroma; necrosis was absent, and the mitotic count was inferior to ½ mm^2^. 

Immunohistochemical examination showed intense staining for desmin (Clone D33 Agilent Technologies Inc., Santa Clara, CA, USA). A diagnosis of mesenchymal neoplasm with smooth muscle differentiation without apparent signs of atypia was given, and the patient was proposed for radical surgical excision of the mediastinal mass.

Surgery was performed through a left lateral muscle-sparing thoracotomy on the fourth intercostal space. At the pleural cavity exploration, the tumor presented as a round-shaped mass with regular margins and a maximum diameter of about 8 cm, adhering to the right upper lobe of the lung and the mediastinal structures at the level of the aortopulmonary window. After adhesiolysis on the upper mediastinal lung surface showing a complete cleavage plane between the tumor and the right upper lobe, the most critical tight adhesions of the mass were found with the pulmonary artery and the aortic arch. The neoplasia was then progressively separated from the pulmonary artery’s wall and the aortic arch by gentle dissection and subsequently radically resected. 

**Figure 3:**
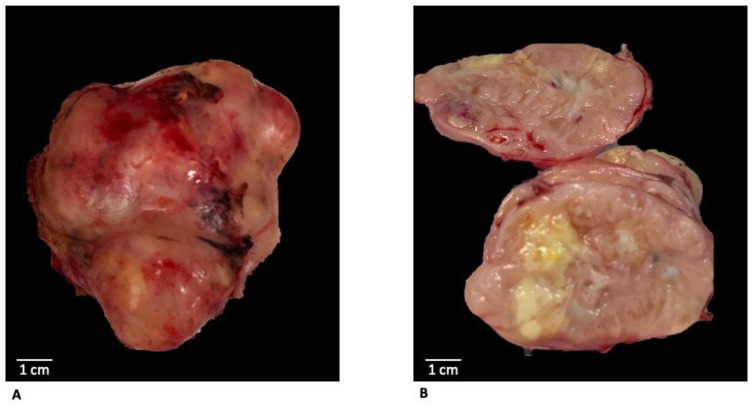
Macroscopic examination after surgical excision showed a large polylobate irregularly shaped mass (**A**) tan to brown with a sharp margin and smooth profile; the cut surface (**B**) showed a whorled pattern admixed with yellowish areas of necrosis and areas of hemorrhage.

A large polylobate irregularly shaped mass tan to brown was revealed on macroscopic examination; the cut surface showed a whorled pattern admixed with yellowish areas of necrosis and areas of hemorrhage (Figure 3). 

**Figure 4:**
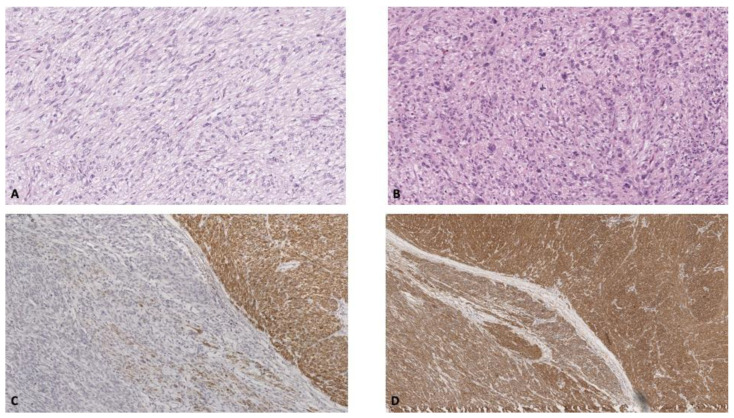
Histologic examination showed alternating areas of (**A**) bland spindle-shaped cells devoid of atypia and higher-grade foci (**B**) characterized by prominent nuclear pleomorphism and atypical mitosis. Desmin immunoperoxidase stain was irregularly conserved across the tumor, mostly lost on higher grade areas (**C**); on the contrary, smooth muscle actin staining was diffuse and intense (**D**).

Microscopic examination showed low-grade spindle cell areas previously assessed on core biopsy with the addiction of higher-grade foci characterized by prominent nuclear pleomorphism, brisk mitotic activity encompassing atypical mitosis (13–15/1.7 mm^2^), and large areas of necrosis (<50% of the whole tumor). The immunophenotypic evaluation was not different from the biopsy sample except for higher grade zones losing cytoplasmic staining for desmin while retaining smooth muscle actin (Clone 1a4 Agilent Technologies Inc., Santa Clara, CA, USA) staining (Figure 4).

Neoplastic cells proliferative index (Clone MIB-1Agilent Technologies Inc., Santa Clara, CA, USA) was high, with hotspots in less differentiated areas of 55–60%. According to these parameters, a grade 2 primary mediastinal leiomyosarcoma diagnosis was given.

## 3. Discussion

Primary mediastinal leiomyosarcomas are rare tumors [1] that, although uncommon, have been consistently reported in the posterior mediastinum but are incredibly scarce in the anterior mediastinum, with less than 10 cases reported in the literature to our knowledge [2,3,4,5,6,7,8].

The most important prognostic factors are dimensions, tumor location, and histologic grade. Leiomyosarcomas of the retroperitoneum and abdominal cavity are the most common subgroup associated with an aggressive clinical course. Leiomyosarcomas of somatic soft tissue are the second most frequent subgroup and are associated with a better prognosis and are thought to arise from small vessels. Even though such lesions could be referred to as “vascular leiomyosarcomas”, this designation usually refers to the third and less common subgroup of tumors arising from medium-size or large veins defined as leiomyosarcomas of vascular origin.

Despite the rarity of these lesions beneath the main challenge in this particular and hard-to-reach anatomical location, resides in the correct grading of the lesions. Primary surgical excision of the lesions, which has been the main and only choice in the past, is only an option at the present time, due to the neoadjuvant chemotherapy and radiotherapy regimens proposed [9]. Downstaging and surgical outcome improvements are the primary aims of these schemes whose effectiveness relies on accurate biopsy grading of the lesions. Core needle biopsy remains the diagnostic tool of choice for deep-seated soft tissue sarcomas due to the general lack of severe complications and accuracy of the CT-guided biopsy. Nevertheless, limited biopsies are sometimes insufficient to reveal useful architectural, immunohistochemical, or molecular features for a definite diagnosis [10]. The distinction between benign and malignant [11] is crucial and needed for surgical planning of excision margins, the feasibility of surgery alone, and consideration of possible neo-adjuvant chemotherapy. A PET/CT scan at time of diagnosis was not available. Literature reports of PET/CT scans of leiomyosarcoma concern mainly abdominal lesions. A PET/CT scan could have helped much in the diagnosis showing a high degree of heterogeneous metabolism. To further comprehend this unusual condition, a PET/CT scan might assist in identifying benign from malignant tumors and in determining the degree of malignancy [12,13]. Quantitative values are expected to be helpful instruments, but no actual assessment has been undertaken due to the limited number of primary tumors studied in this anatomical region. Unfortunately, there are currently no recognized risk models for malignancy in anterior mediastinal masses, making quantitative risk measurement impossible. Moreover, conversely to primary leiomyosarcoma from other anatomical locations, routine use of PET-CT to determine the malignant potential of an anterior mediastinal mass has been questioned in some studies [14] due to good sensitivity but poor overall specificity of the imaging technique.

Tumor grading is also of great relevance for clinical planning and consideration and choice of chemotherapy when surgery is not feasible or the patient has already distant metastases at the time of diagnosis. Tumor heterogeneity and intrinsic variability are crucial in reporting a correct grading [15], even though core needle biopsies may be inadequate and unlikely to be representative of the tumor as a whole, often resulting in a low grade (grade 1) designation, as our case report demonstrates.

As mentioned above, histology from core biopsy undergrads leiomyosarcoma due to undersampling of tumor necrosis. To overcome this problem and to optimize biopsy, radiologists usually avoid targeting necrotic areas, yet to avoid underestimation, identifying high-grade areas in image-guided biopsy is essential. Correlation with radiologic findings (i.e., necrosis assessment) may ameliorate, without definitely resolving, the core biopsy biased view. A Novel approach suggested by Mcaddy [16] and Machado [17]) suggested using modified grading systems encompassing both ki67 and radiologic assessments of necrosis. 

Unfortunately, karyotypic analyses of leiomyosarcomas show complex numeric and structural abnormalities without consistent losses or gains, making no use of diagnostic molecular pathology techniques [18].

Soft tissue sarcomas represent a challenging diagnostic group of tumors due to their location, morphologic spectrum, and unique molecular background. Our case of primary mediastinal leiomyosarcoma is no different from other cases reported although, shows how tumor heterogeneity and limited tissue sampling impact diagnostic entities and challenges an integrated clinical, radiological and histological approach for a more accurate diagnosis. Diagnostic issues of grade reporting can alter the correct clinical handling of patients and, in some cases, could lead to improper treatment/therapies. In this view, ancillary techniques such as molecular pathology could narrow the gap between limited tissue and precise diagnosis: unfortunately, this is not the case for soft tissue leiomyosarcoma. Further studies could shed light on the disease by finding an appropriate molecular signature for each leiomyosarcoma subgroup, providing a more precise diagnosis and the correct background for tailored therapy.

## Data Availability

The data presented in this study are available on request from the corresponding author. The data are not publicly available due to the privacy policy of the centers involved in the study.
